# 

**Published:** 1886-08-20

**Authors:** 


☞ Certain of our subscribers are again reminded that they are in arrears for subscription to the RECORD. Friends attend to this matter at once. Medical journals cannot be run without money. Don't put it off. We need your help and need it now. Week by week we have paid the printers for your journal out of our own pocket, and have not failed to do our part in laboring to make it a good journal for your benefit; and we now ask you to do *your part.* This is your simple duty as an honest man and as a medical gentleman. Let us hear from you at once.

R. C. WORD, M.D., *Managing Editor.*

R. C. WORD, M. D., Managing Editor.

### EDITORIAL NOTICES.

**Something Useful.**—To any one who will send us four cash subscribers, we will send as a premium one of Barry's splendid Chemical Thermometers.

R. C. WORD, *Managing Editor.*

☞ **For Sale**.—A scholarship of Business College at Louisville, Ky., at reduced rates. Inquire of R. C. Word, Managing Editor of this journal.

**The Southern Medical College**, Atlanta, Ga.—This is one of the best Institutions for medical instruction in the United States. The Chairs are well and ably filled. The curriculum is complete. The building is commodious and well arranged. The dissecting room is admirably fitted up for the purpose. The College Hospital is in good running order, and every facility exists for imparting a thorough medical education. Expenses of board, tuition, etc., very moderate. For futher information address, W. P. NICOLSON, M. D., Atlanta, Ga.

CORRECTION.—In referring to Dr. Gaston's contributions to the "Reference Hand-book," in our notice of that work last month, an error occurred in the titles, which should be corrected and read as follows: Cholecystectomy, Cholicystotomy, and Duodeno-Cholecystostomy.

CAUTION.—When we advertise for parties who are prompt and courteous in business transactions, we regard them as honorable and reliable men, and we have faith in their preparations as being what they claim for them. If, on the other hand, we find a party neglectful of correspondence and indifferent to the rights and gentlemanly courtesies due to others, we set them down as at least doubtful men, and regard their preparations accordingly. We regret, for reasons of this kind, to have to say that we have lost confidence in "Goodwin's Syrup of The Hypophosphites," gotten out by a house in Macon, Ga.

THE ALABAMA MEDICAL AND SURGICAL JOURNAL is a new enterprise just inaugurated by Drs. J. D. S. Davis and W. E. B. Davis. The first number presents a very neat and creditable appearance, and shows marks of ability and enterprise on the part of the editors. It is published in Birmingham, Ala. Success to you, gentlemen, in the arduous but useful enterprise upon which you have entered.

PHILLIPS' PHOSPHO MURIATE OF QUININE.—We are in receipt of a sample of this preparation from the Charles H. Phillips Chemical Company, New York. It is a combination of the Wheat Phosphates (Potass Magnesia, Lime and Iron) with Muriate of Quinine and Strychnia. We are very favorably impressed with the formula of this preparation, which, indeed, cannot fail to impress favorably every practitioner, especially in malarious districts. This house has an advertisement on first outside page of our journal.

LITHIA SPRING—Be sure to read the advertisement of the Lithia Spring, or "Salt Spring" as usually called in this city. It is becoming a very popular resort. The water are daily sold on our streets, and great and wonderful accounts are given of its medicinal virtues.

SCHUMANN'S PHARMACY.—We invite special attention to the advertisement of Schumann's Pharmacy. Mr. Schumann's is an old and well established house in this city. He is regarded as a thorough Pharmacist, and is a prompt and active business man. His clerks are polite and attentive. He gives special attention to prescriptions, which are prepared with great neatness and precision. He keeps a full stock of Chemicals, etc., always on hand.

OUR ADVERTISEMENTS.—Be sure to notice the advertisements. They contain much interesting and instructive matter, and they cost the reader nothing, while they are very important to the success of the journal. Our reading matter fills a definite number of pages, and is not allowed to be encroached upon by the advertisements. Not knowing this fact, subscribers sometimes make the mistake of supposing that the space given to advertising is taken from the body of the journal. Not so with the RECORD. So that a large number of advertisements in no degree detracts from the journal, but adds to its interest. It evinces confidence on the part of advertisers, and gives assurance of the success and prosperity of the journal.

